# Scribe Impacts on Provider Experience, Operations, and Teaching in an Academic Emergency Medicine Practice

**DOI:** 10.5811/westjem.2015.6.25432

**Published:** 2015-10-20

**Authors:** Jeremy J. Hess, Joshua Wallenstein, Jeremy D. Ackerman, Murtaza Akhter, Douglas Ander, Matthew T. Keadey, James P. Capes

**Affiliations:** * Emory University School of Medicine, Department of Emergency Medicine, Atlanta, Georgia; † Massachusetts General Hospital, Department of Emergency Medicine, Boston, Massachusetts

## Abstract

**Introduction:**

Physicians dedicate substantial time to documentation. Scribes are sometimes used to improve efficiency by performing documentation tasks, although their impacts have not been prospectively evaluated. Our objective was to assess a scribe program’s impact on emergency department (ED) throughput, physician time utilization, and job satisfaction in a large academic emergency medicine practice.

**Methods:**

We evaluated the intervention using pre- and post-intervention surveys and administrative data. All site physicians were included. Pre- and post-intervention data were collected in four-month periods one year apart. Primary outcomes included changes in monthly average ED length of stay (LOS), provider-specific average relative value units (RVUs) per hour (raw and normalized to volume), self-reported estimates of time spent teaching, self-reported estimates of time spent documenting, and job satisfaction. We analyzed data using descriptive statistics and appropriate tests for paired pre-post differences in continuous, categorical, and ranked variables.

**Results:**

Pre- and post-survey response rates were 76.1% and 69.0%, respectively. Most responded positively to the intervention, although 9.5% reported negative impressions. There was a 36% reduction (25%–50%; p<0.01) in time spent documenting and a 30% increase (11%–46%, p<0.01) in time spent in direct patient contact. No statistically significant changes were seen in job satisfaction or perception of time spent teaching. ED volume increased by 88 patients per day (32–146, p=0.04) pre- to post- and LOS was unchanged; rates of patients leaving against medical advice dropped, and rates of patients leaving without being seen increased. RVUs per hour increased 5.5% and per patient 5.3%; both were statistically significant. No statistically significant changes were seen in patients seen per hour. There was moderate correlation between changes in ED volume and changes in productivity metrics.

**Conclusion:**

Scribes were well received in our practice. Documentation time was substantially reduced and redirected primarily to patient care. Despite an ED volume increase, LOS was maintained, with fewer patients leaving against medical advice but more leaving without being seen. RVUs per hour and per patient both increased.

## INTRODUCTION

Patient care includes a range of indirect activities, such as reviewing patient charts, documenting findings and impressions, ordering and reviewing tests, and interacting with other healthcare personnel. Indirect care constitutes a significant proportion of emergency medicine (EM) physician tasks,^1^ and was found to occupy more than half of EM physicians’ time in academic settings in one study.^2^

Scribes–paraprofessional staff that perform charting and sometimes other tasks for licensed medical providers–have been used to reduce indirect patient care demands. Scribes have long been a fixture in American healthcare^3^,^4^ but have become more common in the last decade. Their roles are generally agreed upon but not fixed. Their primary role is to document in the medical record at the direction of a physician. Scribes can also help navigate the medical record, gather results of laboratory and radiographic testing, and assist with managing and coordinating communication with consulting and referring physicians.

Scribes have become common in EM, and scribe services are typically acquired through contracts with national corporations. Scribe corporations tout the benefits of scribes for emergency departments (EDs),^5^–^7^ citing known associations between waits and delays in care and patient satisfaction and quality of care,^8^,^9^ as well as associations between physician job satisfaction and time for teaching in academic settings^10^–^12^ and links between job satisfaction and burnout risk, which is particularly high in EM.^13^ Corporations also highlight the potential impacts on the bottom line by increasing the number of patients seen per hour and improving documentation, reducing downcoding and thereby increasing reimbursement.

Scribes have recently become a significant part of the healthcare landscape in recent years and have been seen, in part, as a workaround for cumbersome electronic medical records.^14^ Research on scribes and their impacts on EM is growing. Preliminary work in the form of recently published abstracts has substantiated some of scribe service providers’ claims, suggesting, for instance, that scribes have the potential to protect against burnout,^15^ that scribe services may increase ED throughput,^16^ productivity among certain providers,^17^ and revenues,^18^ decrease turnaround time for billing,^17^ and decrease downcoding.^18^ Other work suggests that scribes can increase the amount of teaching in a clinical shift.^19^

Several published studies suggest scribe programs have the potential to improve EM productivity and operations but that improvements vary by context. Arya et al. found that at one-year post-implementation of a scribe program there was an increase in patients seen per hour and in relative value units (RVUs) generated per hour but no effect on time to discharge.^20^ Marshall et al. found no change in patients seen per hour, a decrease in patient length of stay (LOS), and no change in physician charges.^21^ Bastani et al. found post-scribe improvements in the time to see a provider and the time from provider to admission as well as in ED LOS and patient satisfaction.^22^ Walker et al. found a decrease in time to provider and increase in productivity and revenue in their Melbourne ED.^23^ Using retrospective methods, Allen et al. found an increase in ED throughput and provider satisfaction after scribes were implemented.^24^

While scribe impacts on productivity have been studied prospectively, research on other outcomes such as provider satisfaction and teaching have been retrospective. The goal of this prospective study was to assess a scribe program’s impact on ED throughput, physician time utilization, and physician job satisfaction in a large, urban, academic EM practice. Our hypothesis was that the incorporation of a scribe program would increase the amount of time spent in direct contact with patients, increase the amount of time spent teaching students and residents, improve overall work efficiency, and improve provider job satisfaction.

## METHODS

### Study Setting and Design

This was a prospective quasi-experimental pre-post design conducted in an academic EM practice supporting multiple EDs. The scribe program was implemented in two of these EDs, both in primary teaching hospitals within our university medical center with a combined volume of 100,000 annual patient visits. Our EM academic practice has approximately 70 providers working at these two sites and an annual turnover rate of approximately 3%. Providers typically work in one of the two sites as well as an independently-owned county hospital that did not implement a scribe program. Both scribe sites host residents and medical students.

### Selection of Participants

Study subjects were EM physicians with clinical and teaching responsibilities in our academic practice. Physicians were eligible if at least half of their clinical time was spent at one of the two scribe sites (hereafter termed primary site). There was no minimum clinical time threshold required to participate. The study was approved by our institutional review board and participants gave written consent to participate.

### Interventions

The intervention was the implementation of a scribe program at the two clinical sites. Emergency Medical Scribe Systems (EMSS) implemented the program and provided ongoing program management. There were no financial arrangements between EMSS and any of the authors. In the EMSS model, scribes are college students or recent college graduates interested in health science careers. Scribes receive on the job training and are considered by EMSS to be proficient after 15 shifts and skilled after 45 shifts. The program was initiated in January 2012 and fully staffed (defined as greater than 95% of shifts with a scribe) beginning April 2012. Scribes and providers are matched for a shift and the scribe works closely with the physician and transcribes the history of present illness, physical exam findings, differential diagnosis, and medical decision-making. The scribe also documents orders, procedures, test results, and consultant input, as well as patient re-evaluations and final disposition. Scribe charts are forwarded to providers for review, amendment, and signature. By the time post-intervention data were collected all scribes had enough experience to be considered skilled (i.e. each had worked more than 45 shifts).

### Methods and Measurements

This study used multiple data collection methods. The primary sources of data were administrative data and two self-administered online surveys of EM physicians, one administered prior to the scribe intervention and one approximately six months afterward.

The administrative data were collected for similar time periods and included data on ED throughput, including ED LOS, defined as the time between patient arrival and departure from the ED; the rate of patients leaving without being seen (WBS) and leaving against medical advice (AMA). These metrics are standardized across the specialty^25^ and known to correlate with other outcomes such as ED crowding and wait times.^26^ Data on provider productivity including patients seen per hour, RVUs per hour, and RVUs per patient were collected through administrative records. Pre- and post-intervention data were collected for four months each. We analyzed both raw and normalized productivity data; normalized data were generated by dividing monthly provider-specific data by monthly site-specific ED volume.

No validated survey instrument was available so surveys were drafted using a logic model of provider satisfaction and charting activities, tested on a convenience sample of faculty, and revised according to their input. Surveys were self-administered anonymously online. Provider-descriptive data was obtained, including hospital site, cumulative time in the academic practice, and clinical time commitment at the primary site. Self-reported information included uncompensated time spent charting after a shift, job satisfaction, and estimates of time spent in various clinical teaching activities. The pre-intervention survey collected information regarding expectations of scribe program impacts on charting and other activities, and the post-intervention surveys collected information regarding scribe activities and impressions of scribe program impacts. Questions were a mix of categorical and ordinal variables including Likert scales and continuous variables; some variables were recoded for analysis. The survey instruments are included as supplementary material.

### Outcomes

We evaluated multiple outcomes for each line of inquiry. For ED throughput, change in ED LOS pre- to post-intervention was the primary outcome, and pre- to post-intervention changes in the rate of patients leaving WBS and AMA were secondary outcomes. For provider productivity the primary outcome was average provider-level pre-to-post change in monthly average RVUs per hour, and average provider-level changes in monthly average patients per hour and RVUs per patient were secondary outcomes. We evaluated these changes for the entire sample and stratified by site. For teaching, the primary outcome was pre-to-post changes in self-reported estimates of time spent teaching residents and medical students, and changes in time spent teaching at the bedside for both learner types were secondary outcomes. For provider experience, the primary outcomes were pre-to-post changes in self-reported estimates of average time spent charting after a shift and self-reported job satisfaction. Secondary outcomes were self-reported estimates of impacts on charting and pre-to-post changes in time spent charting outside the ED.

### Analysis

We performed data analysis using SPSS version 20 (SPSS Inc., Chicago, IL). ED throughput data for each site were aggregated by month and monthly values were compared pair-wise for each site and in aggregate. We compared the significance of observed differences using paired-sample T-tests for continuous variables and chi-square tests for categorical variables. Provider productivity data were de-identified and monthly pair-wise comparisons for each provider were made in aggregate and stratified by site and evaluated using paired-sample T-tests. Survey data were analyzed using descriptive statistics and pre- to post-intervention changes in primary and secondary outcomes for categorical variables were compared using chi-square tests and for ordinal variables were compared using Wilcoxon signed-rank tests. Differences were reported as point estimates with 95% confidence intervals. Statistical significance was determined at the α=0.05 level.

## RESULTS

Seventy-four faculty members were eligible to for the pre-intervention survey and 71 for the post-intervention survey. The pre-intervention survey response rate was 76.1%, and the post-intervention survey response rate was 69.0%. The main characteristics of the respondent groups and differences between the pre- and post-intervention survey groups are listed in Table 1.

The average monthly clinical workload in the pre- and post-groups sampled was 56 hours and 52 hours, respectively, with a p of 0.58 for the difference. There was a significant pre-to-post shift from dictation to relying on scribes to document patient encounters.

### Provider Perceptions of Scribe Activities

Providers’ impressions of the activities scribes performed demonstrated that scribes most consistently documented physical exams, test results, and discussions with family and other providers. Providers felt that scribes were less consistent in checking on test progress and documenting procedures by other providers, and that they rarely alerted providers regarding chart underdocumentation, prompted for critical care billing, or assisted with medication reconciliation.

### General Perceptions of Intervention Impacts and Provider Experience

In the pre-intervention survey there was a bimodal distribution in job satisfaction, with a small subset reporting low job satisfaction and a larger subset reporting high satisfaction. Post-intervention there was a higher proportion of “very satisfied” responses, but the changes were not statistically significant (p=0.09).

In general, providers enjoyed working with scribes, with 61.9% of respondents stating that they “liked” or “loved” working with scribes in the post-intervention survey. Of those responding, 73.8% reported an overall positive or very positive attitude toward the scribe intervention, and 64.2% stated that they would be moderately or very disappointed if they could no longer work with scribes in the ED. The acclaim was not universal, however, as 9.5% of respondents had very negative or negative perceptions of the intervention and 14.3% of respondents stated they would not be disappointed at all if not able to work with scribes going forward.

More specifically, providers largely reported a positive impact on their charting efficiency, accuracy, and completeness with the most positive change being attributed to charting efficiency; 82% claimed “positive” or “very positive” changes to their efficiency, while less than 9% stated “negative” or “very negative” effects on their efficiency. The most tepid effect was on chart accuracy, with just over 54% of providers claiming that scribes positively or very positively affected their accuracy, whereas 25% felt that scribes negatively or very negatively impacted their chart accuracy.

Additionally, providers almost unequivocally felt that the scribe intervention freed up more time to teach and to spend with patients and also (in their eyes) improved patient satisfaction. In particular, 60% of providers felt that scribes positively or very positively affected their teaching time and 76% felt similarly about the scribes’ effects on their ability to spend time with patients. Sixty percent thought scribes positively or very positively improved patient satisfaction. Notably, while some providers felt that scribes had no effect in one or more of these areas, no providers all thought that scribes had a negative or very negative effect on time for teaching, time with patients or patient satisfaction.

### Pre-post Intervention Changes in Time Spent with Patients

**Figure 1:**
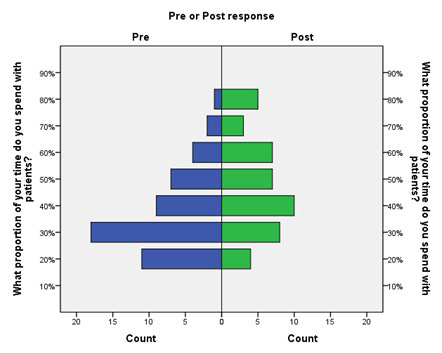
Pre- and post-intervention frequency distributions of reported proportion of shift time spent with patients.

There was a statistically significant pre-to-post increase in the amount of time providers reported spending face-to-face with patients (Figure 1). The weighted average of self-reported time spent with patients went from 37% pre-intervention to 48% post-intervention, an absolute increase of 11% (4%–17%, p<0.01) and a relative increase of 30% (11%–46%).

### Pre-post Intervention Changes in Teaching Medical Students and Residents

Post-intervention, respondents indicated that scribes positively affected their teaching and evaluation habits with both medical students and residents. Forty-two percent of faculty reported spending more time in bedside teaching of medical students, and 28% reported spending more time in bedside teaching of residents. Thirty-three percent of faculty noted they gave more verbal feedback to medical students, and 40% noted they gave more verbal feedback to residents.

Reported changes in the frequency of certain teaching activities bore out some of these perceptions but contradicted others. Regardless, nearly all reported changes were of a small magnitude and not statistically significant. With medical students there was a slight trend toward longer discussion of individual cases and likelihood of teaching at the bedside, but neither change was statistically significant. There was a significant increase in likelihood of giving feedback and identifying specific learning objectives after patient presentations (p<0.01; results not shown). With residents there were drops in the likelihood of seeing patients at the bedside, length of case discussions, and length of time spent giving verbal feedback, none of which was significant. Time spent teaching residents at the bedside showed a nonsignificant increase, and there was no perceptible difference in likelihood of suggesting specific learning objectives for residents (results not shown).

### Pre-to-post Intervention Changes in Time Spent Charting

**Figure 2:**
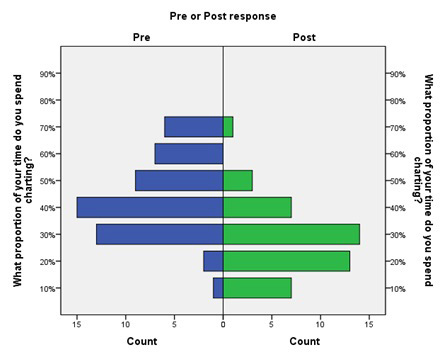
Pre- and post-intervention frequency distributions of reported proportion of shift time spent charting.

In general, post-intervention providers reported spending considerably less time documenting both during and after shifts. In particular, there was a statistically significant decrease in the percent of time spent documenting on-shift (Figure 2; p<0.01). Respondents reported spending a weighted average of 44% of their time charting pre-intervention and 28% post-intervention, for an absolute reduction of 16% (11%–22%, p<0.001) and a relative reduction of 36% (25%–50%).

Respondents also generally reported a lower frequency of leaving charts undone at the end of their shifts, although this result was not statistically significant (p=0.23). There was a statistically significant increase in the proportion of respondents signing charts at the end of their shifts (results not shown; p=0.01). Respondents also reported reductions in the time spent documenting in the ED and outside the ED after shifts but these differences were not statistically significant (p=0.29 and p=0.12, respectively).

### ED Throughput

Changes in ED throughput metrics for each site are presented in Table 2, which presents data aggregated for the entire four-month pre- and post-intervention periods; trends were similar for monthly data at each site. Year-on-year volume increased over the study period at both sites in the range of 3–6%. The rate of patients leaving WBS increased at each site, and the changes at both sites were both operationally and statistically significant. The rate of patients leaving AMA, however, dropped at each site, again at magnitudes that were operationally as well as statistically significant. Patient LOS increased marginally at each site, but the increases were neither operationally nor statistically significant.

### Productivity

Monthly pair-wise changes in raw and normalized productivity metrics for the entire practice are presented in Table 3. Data for individual sites exhibited similar trends. Generally, there was a pre-to-post increase in provider productivity across all metrics in the range of just over 5%. All increases in raw and most increases in normalized RVU/hr were statistically significant, while raw and normalized increases in RVU/pt achieved statistical significance only in certain months. Increases in raw and normalized patients/hour were not statistically significant, although all data showed a consistent trend towards more patients/hour.

## DISCUSSION

Scribes were well received at our sites and resulted in less time charting after shifts, more time spent at the bedside with patients, and more time spent teaching medical students and residents. The intervention was associated with increases in productivity, largely through increased RVUs per patient encounter, and a decreased rate of patients leaving AMA. The scribe program seemed to positively impact all of the core activities of our academic EM practice and was a strategic investment from a management perspective. In general our findings seem in accord with previous literature, although there are some noteworthy differences between our findings and those of prior studies.

For instance, Arya et al. found that, one year post-implementation, for every hour spent with a scribe, providers increased their RVUs/hr by 0.24 and their patients/hr by 0.08.^20^ Full scribe utilization would thus result in increases of 2.4 RVUs/hr and 0.8 patients/hr. We documented a less dramatic productivity increase at our sites six months into the scribe intervention, with an average increase of 0.31 RVUs/hr and 0.1 patients/hr, respectively.

The reason for the difference in magnitude is unclear, though there are several possibilities. First, we evaluated the intervention at our site after only six months and it is possible that full increases in productivity had yet to be realized. Second, it is possible that differences in the scribe programs are partly responsible. Third, ED crowding at our sites constrains patient throughput and did not allow us to take full advantage of the extra leverage that scribes can provide. This is reflected in our left WBS rates that did not fall, yet our AMA rate declined. Once a patient had contact with the MD/scribe team they were more likely to complete their ED care. Given that the number of patients seen per hour increased so modestly at our sites, it is likely that throughput factors were dominant, and that if throughput could be increased to the degree possible at Arya et al.’s site we may have observed similar increases in RVUs/hr.

Marshall et al. found an average decrease in ED LOS of 14.4 minutes and an increase in throughput of 0.28 patients/hr.^21^ Only an abstract is available, which limits comparisons. At our sites we saw a non-statistically significant increase in ED LOS of 8.4 minutes and throughput increase of 0.1 patients/hr and observed an average increase of 0.15 RVUs/patient. Again, the reason for the differences is unclear, although the above-mentioned throughput constraints were likely at least partially responsible for the different observations regarding throughput. The comparison of physician charges is difficult without additional information, but may again result from differences in scribe programs at different sites or be the result of different charting and/or billing practices in the two study settings. Additionally, as several outcomes such as ED LOS are multifactorial,^27^ it is possible that other factors known to affect these measures exert differential influence at specific sites.

Bastani et al. evaluated scribe impacts on ED throughput and patient and provider satisfaction.^22^ At their community site, scribes were implemented shortly after computerized-physician order entry (CPOE), which had worsened ED throughput;^28^ scribes were an attempt to address these deficits. Evaluating the scribe program roughly three months after implementation, they found that the scribe program returned their flow metrics to the pre-CPOE baseline and, for certain metrics (time from seeing a provider to being admitted, LOS for admitted and discharged patients), there was an improvement beyond the baseline. Compared with their pre-CPOE baseline, LOS declined by 13 minutes for admitted patients (2.9%) and 14 minutes for discharged patients (4.9%). This occurred alongside an increase in ED census. It is not clear why their site saw improvements in these ED throughput metrics when ED LOS at our sites increased slightly. Unmeasured differences in the scribe program and/or the study setting are likely responsible.

There is little additional data against which we can benchmark our findings. In two other studies, physicians responded quite positively to scribe programs,^24^,^29^ but the methods used in these studies do not allow direct comparisons. Interestingly, in the Koshy et al. study and ours a non-trivial proportion of providers (approximately 20% and 10%, respectively) did not see scribes as an improvement. Further investigation needs to be done to identify characteristics that might be associated with providers who do not feel their practice is improved by scribes, as our surveys did not bear out clear indications as to why these providers were unhappy with the intervention.

As teaching is central to the mission of academic medical centers, the question of whether scribe programs free up time for clinical teaching activities is an important one. Our results suggest that faculty perceived that the scribe program significantly freed up time for teaching both medical students and residents, but when queried regarding specific teaching activities, the results suggest a more modest impact. A recently published abstract supports the contention that scribes increase teaching time for residents,^19^ though both the structure of the intervention and the outcome studied were different than in our study. Our findings require validation, perhaps via direct observation, to obtain more precise estimates impacts on teaching.

Another important question raised by our findings is how patients perceive scribe interventions and whether scribe programs may increase patient satisfaction. Our respondents felt that patient satisfaction increased, but we did not assess patient responses to the intervention. To the extent that scribes can improve throughput and thereby decrease waits and LOS and free up physician time to improve patient communication and engagement, there is clearly potential for an impact, but this was outside the scope of our study. Future work might explore impacts on patient satisfaction as well.

Finally, there is the question of financial viability of scribe services. While we are not at liberty to share specific financial information regarding the cost of the intervention, the increase in RVU productivity appears to have been adequate to defray the cost of the scribe program going forward.

## STUDY LIMITATIONS

Our study has several potential limitations. First, while the prospective design limits bias, the study is observational and therefore susceptible to influence from various unobserved factors. As process changes are ongoing in every ED, most management interventions do not occur in isolation. During our study period no other significant changes were made. Regardless, we believe the majority of observed impacts are indeed attributable to the scribe program, but it is impossible to determine if some unobserved factors may have biased or confounded the results.

A second potential limitation relates to the use of self-administered surveys, which increases the risk of certain types of bias including non-respondents, recall, and self-interest. Though the response rate was relatively high, non-responders could have significantly differed from responders. There is no way to assess this since the responses were anonymous. Recall bias should have been relatively minor as respondents were asked to report on their practice experience around the time of the surveys. The potential for self-interest bias, which could have resulted in respondents overstating the intervention’s impacts in various areas, is difficult to assess. Additionally, since survey respondents were anonymized, we were not able to pair providers who took both the pre-scribe survey and the post-scribe survey to assess if there were intra-provider attitude changes from scribe implementation. However, the demographics of the two groups (i.e., pre-scribe respondents and post-scribe respondents) were very similar, suggesting the survey respondents per se were very similar pre-to-post. Therefore, we suspect that the aggregate data do reflect, at least to a certain extent, intra-provider effects on attitudes and perceptions of scribes.

A third potential limitation relates to the study time frame and the fact that we assessed faculty response to the intervention relatively shortly after its implementation. We chose this approach to minimize the possibility of bias from other administrative interventions. As faculty were likely still adjusting their practice styles to take full advantage of scribes, however, our findings may be underestimates of true impacts of a mature scribe intervention. As scribe skills mature further and faculty continue to adapt their practices to maximize the potential benefits of working with scribes, we anticipate further improvement in both our throughput metrics and subjective measures.

Finally, our study was limited by the fact that there were no validated instruments available for assessing several of the outcomes we were interested in, and we had to develop and pilot survey-based measures. In most cases our results suggest internal consistency, but the differential shifts in time available for teaching residents and medical students, which theoretically should have shifted in tandem, is difficult to explain and may bring into question the validity of the approach used to measure these outcomes.

## CONCLUSION

Scribes were well received in our academic EM practice, substantially reducing provider charting burdens during and after shifts. Providers reported devoting the time gained to patient care and, to a lesser degree, teaching. The intervention increased provider productivity, primarily the result of increased RVUs per hour and per patient, although it had modest impacts on ED throughput. Findings are largely consistent with prior studies and suggest generally positive impacts on most aspects of academic practice, although some productivity increases may be limited by larger contextual factors. Impacts on teaching and patient satisfaction require validation and future study.

## Figures and Tables

**Table 1:** Main characteristics of the study population, study sites, and pre-post differences with regard to the impact of the use of scribes on provider experience.

	Pre		Post		Sample		
		
	N	%	N	%	N	%	p for χ^2^
Clinical site							
Academic tertiary	26	48.1	21	42.9	47	47.0	0.59
Academic community	28	51.9	28	57.1	56	54.4	
Years in this practice							
0–5	32	59.3	28	57.1	60	58.3	0.83
>5	22	40.7	21	42.9	43	41.7	
Clinical activity (hours per month)							
≤40	16	30.8	24	50.0	40	38.8	0.10
41–80	30	57.7	18	36.7	48	46.6	
>80	6	11.5	7	14.3	13	12.6	
Ever used scribes							
Yes	3	5.6	49	100.0	52	50.5	<0.01
No	51	94.4	0	0.0	51	49.5	
Dictation frequency							
Rarely	39	72.3	45	91.9	84	81.6	0.02
Sometimes	8	14.8	2	4.1	10	9.7	
Frequently	7	13.0	2	4.1	9	8.7	
Total sample	54	100.0	49	100.0	103	100.0	

**Table 2:** Pre-post differences in emergency department flow metrics at each site and for the combined sample.

Site	Metric	2011 Mean	2012 Mean	Difference	% Change	95% CI	p-value
Academic	Total patients*	698.89	720.50	21.61	3.1	−6.1–49.3	0.12
	% Left AMA	1.26	0.92	−0.34	−27.0	−0.7–−0.0	0.04
	% Left WBS	1.50	2.81	1.31	87.3	0.7–1.9	<0.01
	LOS (hrs)	5.69	5.84	0.14	2.5	−0.2–0.4	0.32
Community	Total patients*	1099.39	1166.61	67.22	6.1	31.6–102.9	<0.01
	% Left AMA	1.54	1.28	−0.26	−16.7	−0.5–−0.0	0.04
	% Left WBS	3.85	5.39	1.54	40.0	0.7–2.4	<0.01
	LOS (hrs)	5.15	5.29	0.13	2.5	−0.0–0.3	0.13
Combined	Total patients*	1798.28	1887.11	88.83	5.0	31.8–145.9	0.04
	% Left AMA	1.43	1.14	−0.29	−20.3	−0.52–−0.06	0.02
	% Left WBS	2.94	4.41	1.47	50.0	0.83–2.11	<0.01
	LOS (hrs)	5.42	5.56	0.14	2.6	−0.05–0.33	0.15

*AMA*, against medical advice; *WBS*, without being seen; *LOS*, length of stay

* monthly average values for each period.

**Table 3:** Pre-post differences in seasonally-matched raw and normalized productivity metrics for the combined sample. Raw data are designated with an R and normalized with an N. Pair-wise comparisons could be done for all months for 62 providers.

Metric	Month paired	2011 Mean	2012 Mean	Diff	95% CI	% change	p-value
RVU/hr	September (R)	5.81	6.44	0.32	0.32–0.93	5.51%	<0.01
	September (N)	0.0013	0.0014	0.8×10^−4^	0.1×10^−4^–1.4×10^−4^	8.06%	0.03
	October (R)	5.45	6.00	0.21	0.21–0.88	3.85%	<0.01
	October (N)	0.0015	0.0017	1.6 ×10^−4^	0.7×10^−4^–2.5×10^−4^	13.6%	<0.01
	November (R)	5.49	5.99	0.18	0.18–0.82	3.28%	<0.01
	November (N)	0.0013	0.0014	1.0 ×10^−4^	0.1×10^−4^–1.8×10^−4^	10.2%	0.03
	December (R)	5.48	5.99	0.52	0.22–0.81	9.49%	<0.01
	December (N)	0.0017	0.0017	0.3 ×10^−4^	−0.6×10^−4^–1.1×10^−4^	2.64%	0.57
RVU/pt	September (R)	2.88	3.09	0.21	0.12–0.31	7.29%	<0.01
	September (N)	0.0007	0.0007	0.1 ×10^−4^	−0.1×10^−4^–0.3×10^−4^	1.84%	0.39
	October (R)	2.86	3.07	0.21	0.10–0.32	7.34%	<0.01
	October (N)	0.0008	0.0009	0.7 ×10^−4^	0.3×10^−4^–0.1×10^−4^	7.83%	<0.01
	November (R)	2.91	2.98	0.07	−0.21–0.15	2.41%	0.14
	November (N)	0.0007	0.0007	0	−0.2×10^−4^−0.2×10^−4^	−0.33%	0.98
	December (R)	2.92	3.04	0.12	0.02–0.22	4.11%	0.02
	December (N)	0.0009	0.0009	−0.3 ×10^−4^	−0.6×10^−4^–0.0×10^−4^	−3.45%	0.08
Pt/hr	September (R)	2.05	2.13	0.09	−0.05–0.22	4.39%	0.21
	September (N)	0.0005	0.0005	0	−0.3×10^−4^–0.3×10^−4^	2.41%	0.86
	October(R)	1.92	1.99	0.07	−0.08–0.21	3.65%	0.37
	October (N)	0.0005	0.0006	0.2 ×10^−4^	−0.2×10^−4^–0.5×10^−4^	7.37%	0.36
	November(R)	1.92	2.04	0.12	−0.01–0.25	6.25%	0.71
	November (N)	0.0004	0.0005	0.2 ×10^−4^	−0.1×10^−4^–0.5×10^−4^	8.45%	0.23
	December (R)	1.89	2.01	0.12	−0.00–0.23	6.35%	0.06
	December (N)	0.0006	0.0006	−0.1 ×10^−4^	−0.5×10^−4^–0.2×10^−4^	−0.76%	0.37

*RVU*, relative value units; *Pt*, patient

## References

[1] Perina DG, Brunett CP, 2011 EM Model Review Task Force (2012). The 2011 Model of the Clinical Practice of Emergency Medicine. Acad Emerg Med.

[2] Chisholm CD, Weaver CS, Whenmouth L (2011). A Task Analysis of Emergency Physician Activities in Academic and Community Settings. Ann Emerg Med.

[3] Allred RJ, Ewer S (1983). Improved emergency department patient flow: five years of experience with a scribe system. Ann Emerg Med.

[4] Hixon JR (1981). Scribe system works like a charm in Sarasota ED. Emerg Dep News.

[5] (2009). Expanded scribe role boosts staff morale. ED Manag.

[6] (2009). Scribes, EMR please docs, save $600,000. ED Manag.

[7] Guglielmo WJ (2006). What a scribe can do for you. Med Econ.

[8] Epstein SK, Huckins DS, Liu SW (2012). Emergency department crowding and risk of preventable medical errors. Intern Emerg Med.

[9] Johnson KD, Winkelman C (2011). The effect of emergency department crowding on patient outcomes: a literature review. Adv Emerg Nurs J.

[10] McConville JF, Rubin DT, Humphrey H (2001). Effects of billing and documentation requirements on the quantity and quality of teaching by attending physicians. Acad Med.

[11] McLean SA, Feldman JA (2001). The impact of changes in HCFA documentation requirements on academic emergency medicine: results of a physician survey. Acad Emerg Med.

[12] Embi PJ, Yackel TR, Logan JR (2004). Impacts of computerized physician documentation in a teaching hospital: perceptions of faculty and resident physicians. J Am Med Inform Assoc.

[13] Shanafelt TD, Boone S, Tan L (2012). BUrnout and satisfaction with work-life balance among us physicians relative to the general us population. Arch Intern Med.

[14] George A, Gellert M (2014). The Rise of the Medical Scribe Industry Implications for the Advancement of Electronic Health Records.

[15] Brown L, Benage M, Tran A (2014). 121 Impact of Scribes Upon Emergency Physician Self-Assessed Authenticity. Ann Emerg Med.

[16] Heaton HA, Matthews SM, Cummings AG (2014). 207 Impact of Scribes on Patient Throughput in an Academic Emergency Department. Ann Emerg Med.

[17] Heaton HA, Matthews SM, Cummings AG (2014). 6 Financial Impact of Scribes in an Academic Emergency Department. Ann Emerg Med.

[18] Gupta NJ, Kopp M, Becker BM (2014). 247 Scribes in an Academic Emergency Department Lead to Increased Charges and Decreased Down Coding. Ann Emerg Med.

[19] Wegg B, Deibel M, Kiernan C (2014). 162 Advancing Resident Training With the Use of Scribes. Ann Emerg Med.

[20] Arya R, Salovich DM, Ohman-Strickland P (2010). Impact of scribes on performance indicators in the emergency department. Acad Emerg Med.

[21] Marshall JS, Verdick CM, Tanaka MS (2012). Implementation of Medical Scribes in an Academic Emergency Department: Effect on Emergency Department Throughput, Clinical Productivity, and Emergency Physician Professional Fees.

[22] Bastani A, Shaqiri B, Palomba K (2014). An ED scribe program is able to improve throughput time and patient satisfaction. Am J Emerg Med.

[23] Walker K, Ben-Meir M, O’Mullane P (2014). Scribes in an Australian private emergency department: A description of physician productivity. Emerg Med Australas.

[24] Allen B, Banapoor B, Weeks EC (2014). An Assessment of Emergency Department Throughput and Provider Satisfaction after the Implementation of a Scribe Program. Adv Emerg Med.

[25] Welch SJ, Stone-Griffith S, Asplin B (2011). Emergency Department Operations Dictionary: Results of the Second Performance Measures and Benchmarking Summit. Acad Emerg Med.

[26] Mohsin M, Forero R, Ieraci S (2007). A population follow-up study of patients who left an emergency department without being seen by a medical officer. Emerg Med J.

[27] Hobbs D, Kunzman SC, Tandberg D (2000). Hospital factors associated with emergency center patients leaving without being seen. Am J Emerg Med.

[28] Bastani A, Walch R, Todd B (2010). 253: Computerized Prescriber Order Entry Decreases Patient Satisfaction and Emergency Physician Productivity. Ann Emerg Med.

[29] Koshy S, Feustel PJ, Hong M (2010). Scribes in an Ambulatory Urology Practice: Patient and Physician Satisfaction. J Urol.

